# Regulation of DNA methyltransferase 1 transcription in BRCA1-mutated breast cancer: a novel crosstalk between E2F1 motif hypermethylation and loss of histone H3 lysine 9 acetylation

**DOI:** 10.1186/1476-4598-13-26

**Published:** 2014-02-06

**Authors:** Da Li, Fang-Fang Bi, Ji-Min Cao, Chen Cao, Bo Liu, Qing Yang

**Affiliations:** 1 Department of Obstetrics and Gynecology, Shengjing Hospital, China Medical University, Shenyang 110004, China; 2 Experimental Research Center, Shengjing Hospital, China Medical University, Shenyang 110004, China; 3 Department of Physiology and Pathophysiology, Institute of Basic Medical Sciences, Chinese Academy of Medical Sciences, School of Basic Medicine Peking Union Medical College, Beijing 100005, China; 4 Department of Pathology, Chinese PLA General Hospital, Beijing 100853, China; 5 Department of Laboratory Medicine, No. 1 Hospital of China Medical University, Shenyang 110001, China

**Keywords:** DNMT1, Histone modifications, E2F1, BRCA1, Breast cancer

## Abstract

**Background:**

DNA methyltransferase 1 (DNMT1) plays a critical role in breast cancer progression. However, the epigenetic mechanism regulating DNMT1 expression remains largely unknown.

**Methods:**

Epigenetic regulation of DNMT1 was assessed in 85 invasive ductal carcinomas from BRCA1 mutation carriers. Association between clinicopathological features and DNMT1 promoter methylation was determined using Fisher’s exact test. Univariate analysis of survival was performed using the Kaplan-Meier method. Multivariate Cox regression analysis was performed to identify the independent prognostic factors for overall survival.

**Results:**

Hypermethylated E2F transcription factor 1 (E2F1) motif is a key regulatory element for the DNMT1 gene in BRCA1-mutated breast cancer. Mechanistically, the abnormal E2F1 motif methylation-mediated loss of active histone H3 lysine 9 acetylation (H3K9ac) and transcription factor E2F1 enrichment synergistically inhibited the transcription of DNMT1. Clinicopathological data indicated that the hypermethylated E2F1 motif was associated with histological grade, lymph node, Ki67 and E-cadherin status; univariate survival and multivariate analyses demonstrated that lymph node metastasis was an independent and reliable prognostic factor for BRCA1-mutated breast cancer patients.

**Conclusions:**

Our findings imply that genetic (such as BRCA1 mutation) and epigenetic mechanisms (such as DNA methylation, histone modification, transcription factor binding) are jointly involved in the malignant progression of DNMT1-related breast cancer.

## Background

Breast cancer is the most prevalent malignancy, and is a leading cause of mortality in women worldwide [1]. Accumulating evidence indicates that hereditary factors and epigenetic events are implicated in the initiation and progression of breast cancer and resistance to endocrine therapies [2]. To date, BRCA mutations are the only known cause of hereditary breast cancer [3], and DNA methylation is among the most well-studied epigenetic modifications, which is maintained by the enzyme DNA methyltransferase 1 (DNMT1) [4]. The growing body of research suggests that DNMT1 plays an important role in the development of breast cancer [5-12]. However, expression levels of DNMT1 in breast cancer tissues have been a matter of debate [5,6,8,10,12], and the underlying mechanism is still not entirely clear. In mammals, promoter methylation at CpG dinucleotides is an important feature regulating gene expression [13]. Therefore, the present study was undertaken to investigate the DNA methylation patterns in the core promoter region of DNMT1 in human breast cancers with identified BRCA1 mutations compared to those without, and to provide novel insight into the mechanisms involved in the regulation of DNMT1 expression.

## Results

### BRCA1-mutated breast cancer displayed a hypermethylated E2F1 motif and promoter region

**Figure 1:**
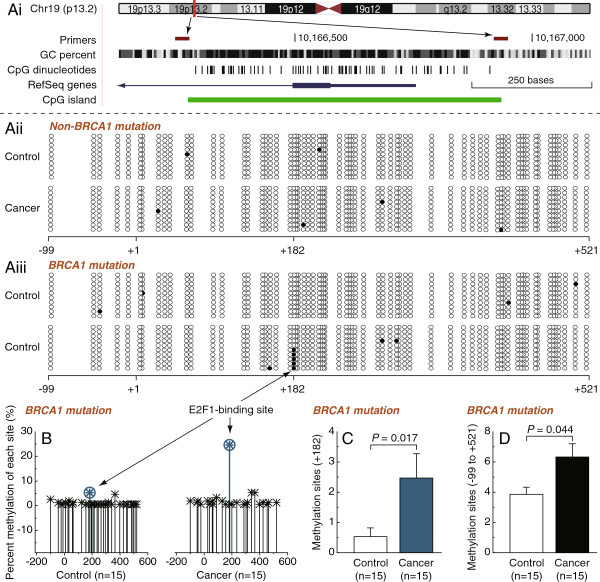
**Methylation patterns of the DNMT1 promoter in BRCA1-mutated and non-mutated breast cancer. Ai**, location of CpG sites in the core promoter region of DNMT1. Genomic coordinates are shown, along with the primer-amplified fragments, GC percentage, location of individual CpG dinucleotides (dashes), the DNMT1 RefSeq gene (exon 1 shown as a blue box and intron shown as an arrowed line), and CpG island (green bar). The arrow indicates the direction of transcription. **Aii and Aiii**, comparative analysis of methylation patterns in the core promoter region of DNMT1 in non-mutated and BRCA1-mutated breast cancer, and their adjacent normal breast tissues (each group, n = 15). The circles correspond to the CpG sites denoted by black dashes in Figure 1Ai. Closed circles, methylation; open circles, unmethylated. Ten individual clones were sequenced for each sample. Arrow shows the methylation of a cytosine located in a CpG within the E2F1 motif (position +182, +1 is the transcription initiation site). **B**, summary of the methylation patterns of DNMT1 core promoter in BRCA1-mutated breast cancer and adjacent normal breast tissues. The y-axis shows the mean methylation sites. **C** and **D**, overall methylation percentage of the E2F1 motif and the DNMT1 core promoter region (-99 to +521) from BRCA1-mutated breast cancer and adjacent normal breast tissues. Bar graphs show mean ± SD.

To investigate DNMT1 transcriptional regulation through epigenetic mechanisms, we assessed the DNA methylation status of the DNMT1 core promoter region (from -99 to +521, with +1 denoting the transcription initiation site) in BRCA1-mutated and non-mutated breast cancer, and their adjacent normal breast tissues. As shown in Figure 1Aiii and 1B, BRCA1-mutated breast cancer exhibited global promoter hypermethylation (*P* = 0.044; Figure 1D), especially around the E2F1 motif (*P* = 0.017; Figure 1C). No similar changes were observed in non-BRCA1-mutated breast cancer (Figure 1Aii).

### Low DNMT1 transcript levels showed a significant inverse correlation with hypermethylated E2F1 motif in BRCA1-mutated breast cancer

**Figure 2:**
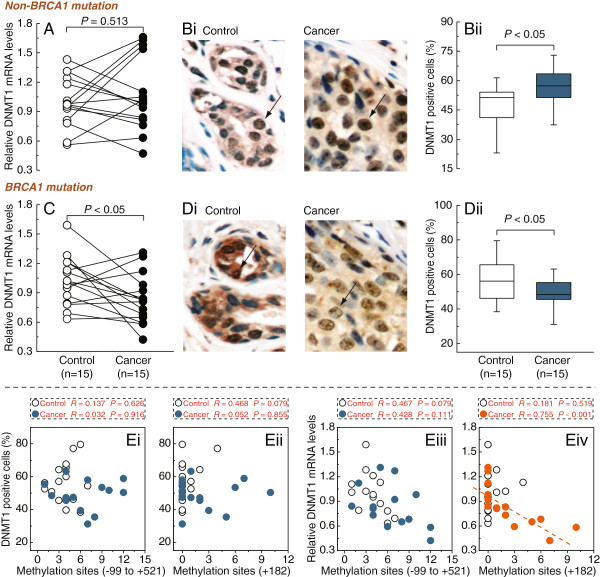
**Expression patterns of DNMT1 in BRCA1-mutated and non-mutated breast cancer. A** and **C**, relative DNMT1 mRNA levels were measured in non-mutated and BRCA1-mutated breast cancer, and their adjacent normal breast tissues. **Bi** and **Di**, sections were subjected to immunostaining for DNMT1 in non-mutated and BRCA1-mutated breast cancer, and their adjacent normal breast tissues. Arrow shows positive staining for DNMT1 in the nuclei. **Bii** and **Dii**, summary of the percentages of DNMT1-positive cells from the measurements shown in **Bi** and **Di**, respectively. Magnification is 400×. **Ei** and **Eii**, correlation between the DNMT1-positive cells and methylated sites (+182) or (-99 to +521) in BRCA1-mutated breast cancer and adjacent normal breast tissues. **Eiii** and **Eiv**, correlation between the relative DNMT1 mRNA levels and methylated sites (+182) or (-99 to +521) in BRCA1-mutated breast cancer and adjacent normal breast tissues. Open circles, normal breast tissues; closed circles, breast cancer tissues (each group n = 15).

Real-time PCR and immunohistochemical analysis showed that the levels of DNMT1 mRNA and protein were decreased in BRCA1-mutated breast cancer, compared to adjacent normal breast tissues (*P* < 0.05; Figure 2C and D), DNMT1 protein levels was further confirmed by western blotting [see Additional file 1]. Although there was no significant difference in mRNA levels between non-BRCA1-mutated breast cancer and adjacent normal breast tissues (Figure 2A, *P* = 0.513), the protein expression of DNMT1 was upregulated (*P* < 0.05; Figure 2B). In addition, we analyzed the correlation between DNMT1-positive cells or mRNA levels and the number of methylated sites within the E2F1 motif (+182) or DNMT1 core promoter region (-99 to +521) in BRCA1-mutated breast cancer and adjacent normal breast tissue (Figure 2Ei-Eiv). It is, however, interesting to note that only a significant inverse correlation was observed between DNMT1 mRNA levels and the methylated E2F1 motif (*R* = 0.755, *P* < 0.001; Figure 2Eiv).

### Hypermethylated E2F1 motif is a key regulatory mechanism for DNMT1 transcription in BRCA1-mutated breast cancer

**Figure 3:**
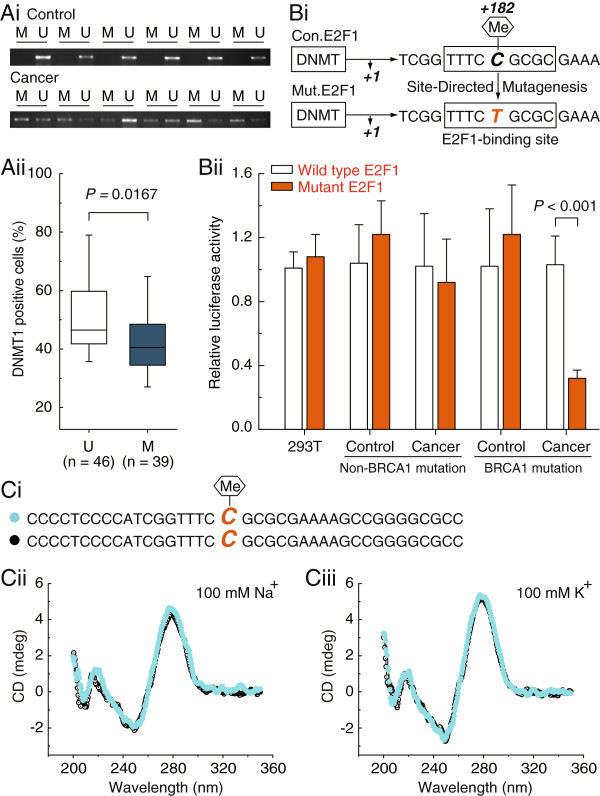
**Repression of DNMT1 promoter activity by methylated E2F1 motif. Ai**, comparative analysis of E2F1 motif methylation between BRCA1-mutated breast cancer and adjacent normal breast tissues. **Aii**, relationship between DNMT1 protein expression and promoter methylation in BRCA1-mutated breast cancer and adjacent normal breast tissues (U; unmethylated group, n = 46, M; methylated group, n = 39). **Bi**, the schematic shows that the nucleotide sequence of consensus E2F1 motif (Con.E2F1) was point mutated at position +182 (C to T) to generate the Mut.E2F1. **Bii**, 293 T cells, and 15 primary non-mutated and BRCA1-mutated breast cancer and their normal breast cells were transfected with Con.E2F1 and Mut.E2F1. 24 hours after transfection, whole-cell extracts were analyzed for luciferase activity. Each experiment was repeated four times for 293 T cells and primary breast cells of each patient. Bar graphs show mean ± SD. **Ci**, the schematic represents the selected nucleotide sequence containing the E2F1 motif, with or without a methyl group at the fifth position of the cytosine pyrimidine ring at position +182. **Cii** and **Ciii**, The CD spectra of the selected nucleotide sequence in the presence of 100 mM Na^+^ or 100 mM K^+^ are shown.

Based on the results above, we next explored the relationship between E2F1 motif methylation and DNMT1 expression in a large number of BRCA1-mutated breast cancer specimens. Similar results were observed using methylation-specific PCR (Figure 3Ai), and BRCA1-mutated cancer specimens were classified into unmethylated and methylated groups for comparison of the protein expression of DNMT1. The methylated group showed significantly lower expression of DNMT1 in comparison to the unmethylated group (*P* = 0.0167; Figure 3Aii). To further confirm the role of cytosine located in the E2F1 motif, a point mutation of cytosine to thymine was inserted in the E2F1 element (Figure 3Bi). Then, the consensus E2F1 (con.E2F1) and mutated E2F1 (mut.E2F1) were transiently transfected into 293 T cells, and primary non-mutated and BRCA1-mutated breast cancer and their corresponding normal breast cells. Notably, only in BRCA1-mutated breast cancer cells was the E2F1 motif found to be the critical element for DNMT1 transcription (Figure 3Bii).

Recently, a substantial body of evidence has been reported which suggests that most of the known genes contain specific motifs, such as G-quadruplex in their promoter regions, which can modulate gene transcription by affecting the binding of histones [14]. Therefore, the CD spectra were used to gain information on whether the methylated E2F1 motif can influence the structure of the DNMT1 promoter. However, our results showed that the methylation of cytosine located in a CpG within the E2F1 motif may not affect the structure of DNMT1 (Figure 3C).

### Loss of H3K9ac and E2F1 enrichment around the hypermethylated E2F1 motif in BRCA1-mutated breast cancer

**Figure 4:**
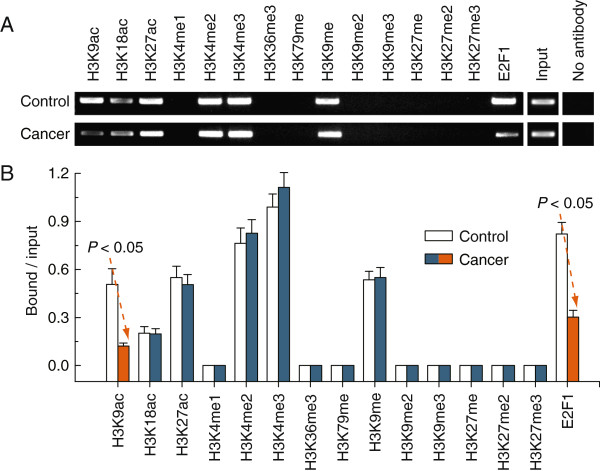
**Comparative analysis of histone modification and E2F1 enrichment around the E2F1 motif between BRCA1-mutated breast cancer and adjacent normal breast tissues. A**, chromatin immunoprecipitation was performed using antibodies to H3K9Ac, H3K18Ac, H3K27Ac, H3K4me1, H3K4me2, H3K4me3, H3K36me3, H3K79me, H3K9me, H3K9me2, H3K9me3, H3K27me, H3K27me2, H3K27me3 and E2F1. PCR was performed for regions within the CpG islands and around the E2F1 motif. A negative control without antibodies is included for comparison. **B**, representative results of 15 primary BRCA1-mutated breast cancer and their normal breast tissues are shown. Bar graphs show mean ± SD.

To obtain further understanding of the regulatory potential of the crosstalk between DNA methylation and histone modification in controlling DNMT1 transcription, we examined the active histone markers H3K9ac, H3K18ac, H3K27ac, H3K4me1, H3K4me2, H3K4me3, H3K36me3 and H3K79me, and the repressive histone markers H3K9me, H3K9me2, H3K9me3, H3K27me, H3K27me2 and H3K27me3 in the core promoter of DNMT1, especially around the E2F1 motif; we also focused on the enrichment of the transcription factor E2F1, due to the fact that the hypermethylated E2F1 motif was observed in BRCA1-mutated breast cancer (Figure 1Aiii). Chromatin immunoprecipitation analysis indicated that the levels of H3K9ac and E2F1 around the E2F1 motif were only significantly decreased in BRCA1-mutated breast cancer (Figure 4A and B). These results, together with the methylation data in Figures 1, 2 and 3, suggest that DNMT1 transcription is associated with changes in epigenetic features, including decreased H3K9ac and E2F1 enrichment, and the hypermethylated E2F1 motif. However, no differences exist in histone markers around the E2F1 motif between normal and cancer tissues in BRCA1 wild type cases [see Additional file 2].

### H3K9ac and E2F1 present in the E2F1 motif are responsible for the transcriptional regulation of DNMT1 in BRCA1-mutated breast cancer

**Figure 5:**
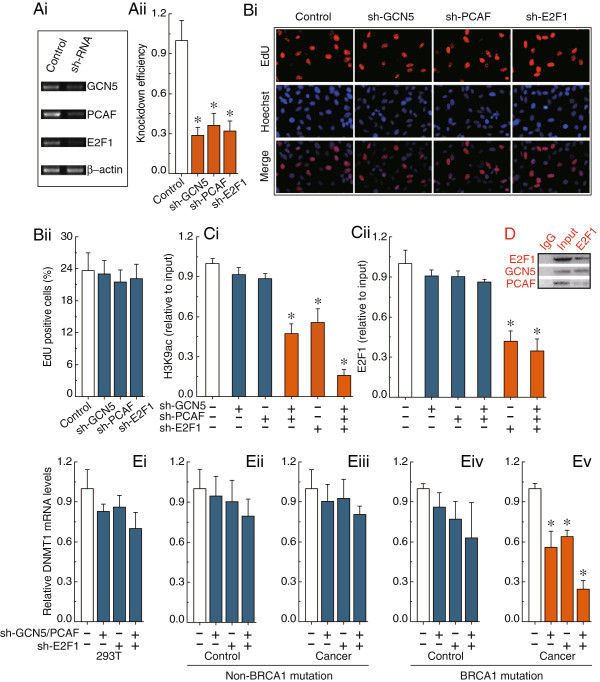
**H3K9ac and E2F1-mediated transcriptional regulation of DNMT1. Ai**, RT-PCR showing GCN5, PCAF and E2F1 levels before and after knockdown by shRNAs, and normalized to β-actin expression. **Aii**, the results from three independent experiments are represented as mean ± SD. **Bi**, EdU labeling showing proliferation of GCN5, PCAF and E2F1-silenced and control cells. Blue, Hoechst 33342 labeling of cell nuclei; Red, EdU labeling of nuclei of proliferative cells. **Bii**, the EdU incorporation rate was expressed as the ratio of EdU positive cells to total Hoechst33342 positive cells. **Ci** and **Cii**, analysis of histone modification H3K9ac and transcription factor E2F1 enrichment around the E2F1 motif within the CpG islands after the deletion of GCN5, PCAF or E2F1. **D**, The interaction of E2F1 and GCN5 or PCAF were examined by the immunoprecipitation of cell extracts with an antibody to E2F1, and co-immunoprecipitation of E2F1, GCN5 and PCAF by western blot analysis. Results of Figure 5A-D were obtained in BRCA1-mutated breast cancer cells, and the same results were also obtained in 293 T cells and non-BRCA1 mutated breast cancer cells. **Ei-Ev**, the DNMT1 expression levels after deletion of H3K9ac and E2F1 around the E2F1 motif in 293 T cells, and 15 primary non-mutated and BRCA1-mutated breast cancer and their normal breast cells. Each experiment was repeated four times for 293 T cells and primary breast cells of each patient. Bar graphs show mean ± SD. * *P* < 0.05 vs. Control.

As described previously by Jin [15], we observed that only the combined knockdown of GCN5 and PCAF can specifically induce a decrease of H3K9ac around the E2F1 motif (Figure 5Ci). Interestingly, knockdown of E2F1 showed a similar effect to knockdown of GCN5 and PCAF (Figure 5Ci); therefore, an alternative possibility may be that E2F1 can recruit GCN5 and PCAF to the E2F1 motif, which was confirmed by co-immunoprecipitation analysis (Figure 5D). As shown in Figure 5Cii, knockdown of E2F1 was an effective way to reduce E2F1 enrichment, but did not change the levels of H3K9ac around the E2F1 motif. Meanwhile, we observed that knockdown of GCN5, PCAF and E2F1 had no detectable effect on the cell morphology and proliferation (Figure 5A and B). Of particular note, as described in Figure 4, the hypermethylated E2F1 motif of the DNMT1 promoter was accompanied by loss of H3K9ac and E2F1 enrichment in BRCA1-mutated breast cancer. After the deletion of H3K9ac and/or E2F1 enrichment in BRCA1-mutated breast cancer, the transcription of DNMT1 was significantly down-regulated (Figure 5Ev).

### Correlation of E2F1 motif methylation with clinicopathological characteristics in BRCA1-mutated breast cancer

The correlation between E2F1 motif methylation and clinicopathological parameters was analyzed using Fisher s exact test. As shown in Table 1, E2F1 motif methylation was associated with histological grade (*P* = 0.006), lymph node (*P* = 0.014), Ki67 (*P* = 0.030) and E-cadherin status (*P* = 0.030). No significant associations were observed between methylation of the E2F1 motif and age at diagnosis, menstrual status, tumor size, estrogen receptor, progesterone receptor, c-erbB-2 or p53 status.

**Table 1:** Association between DNMT1 promoter methylation and clinicopathological features in BRCA1-mutated breast cancer

	***n***	**M**	**(%)**	**UM**	**(%)**	***P***
**Age at diagnosis**						**1.000**
≤ 50 y	30	14	35.90	16	34.78	
> 50 y	55	25	64.10	30	65.22	
**Menstrual status**						**0.282**
Premenopausal	38	20	51.28	18	39.13	
Postmenopausal	47	19	48.72	28	60.87	
**Tumor size**						**0.164**
≤ 5 cm	59	24	61.54	35	76.09	
> 5 cm	26	15	38.46	11	23.91	
**Histological grade**						**0.006**
I-II	63	23	58.97	40	86.96	
III	22	16	41.03	6	13.04	
**LN status**						**0.014**
Positive	35	22	56.41	13	28.26	
Negative	50	17	43.59	33	71.74	
**ER status**						**0.282**
Positive	47	19	48.72	28	60.87	
Negative	38	20	51.28	18	39.13	
**PR status**						**0.269**
Positive	50	20	51.28	30	65.22	
Negative	35	19	48.72	16	34.78	
**c-erbB-2 status**						**0.378**
Positive	36	19	48.72	17	36.96	
Negative	49	20	51.28	29	63.04	
**p53 status**						**0.258**
Positive	30	11	28.21	19	41.30	
Negative	55	28	71.79	27	58.70	
**Ki67 status**						**0.030**
Positive	62	33	84.62	29	63.04	
Negative	23	6	15.38	17	36.96	
**E-cadherin status**						**0.030**
Positive	68	27	69.23	41	89.13	
Negative	17	12	30.77	5	10.87	

M, methylated; UM, unmethylated; LN, lymph node; ER, estrogen receptor; PR, progesterone receptor.

### Multivariate and univariate analysis of overall survival for patients with BRCA1-mutated breast cancer

**Figure 6:**
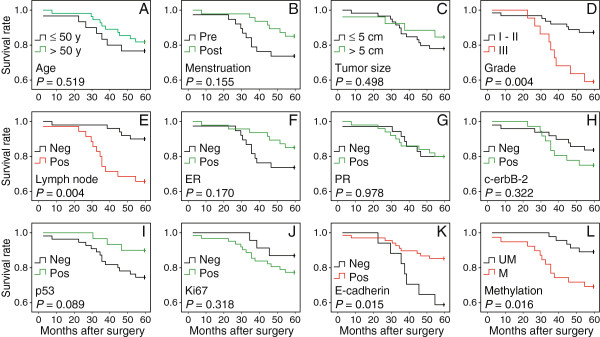
**Kaplan-Meier analysis of overall survival for 85 BRCA1-mutated breast cancer patients.** The following variables were analyzed: age at diagnosis **(A)**, menstruation **(B)**, tumor size **(C)**, grade **(D)**, lymph node **(E)**, ER **(F)**, PR **(G)**, c-erbB-2 **(H)**, p53 **(I)**, Ki67 **(J)**, E-cadherin **(K)** and DNMT1 methylation **(L)**. Pre, premenopausal; Post, postmenopausal; ER, estrogen receptor; PR, progesterone receptor; Neg, negative; Pos, positive; UM, unmethylated; M, methylated.

We analyzed the overall survival to assess the prognostic significance. Multivariate Cox regression analysis indicated that lymph node metastasis was an independent prognostic factor for predicting the overall survival of BRCA1-mutated breast cancer patients (Table 2, *P* = 0.038). We also performed Kaplan-Meier analysis and log-rank tests for overall survival to define prognostic subgroups. The results revealed that the significant prognostic factors were histological grade (Figure 6D, *P* = 0.004), lymph node metastasis (Figure 6E, *P* = 0.004), E-cadherin (Figure 6K, *P* = 0.015) and DNMT1 methylation (Figure 6L, *P* = 0.016). Moreover, patients with premenopausal (Figure 6B, *P* = 0.155), estrogen receptor-negative (Figure 6F, *P* = 0.170) and p53-negative (Figure 6I, *P* = 0.089) breast cancer showed a trend for poor overall survival, although this was not statistically significant. No significant difference in overall survival was found among patients with different ages at diagnosis, tumor size, progesterone receptor status, c-erbB-2 status or Ki67 status (Figure 6A, C, G, H and J).

**Table 2:** Prognostic factors for overall survival by multivariate Cox regression analysis in BRCA1-mutated breast cancer

**Variable**	**RR**	**95%CI**	***P***
**Age at diagnosis** (> 50 y *vs* ≤ 50 y)	1.059	0.982-1.143	0.137
**Menstrual status** (Post *vs* Pre)	0.258	0.043-1.542	0.138
**Tumor size** (> 5 cm *vs* ≤ 5 cm)	0.389	0.107-1.413	0.151
**Histological grade** (III vs I-II)	2.628	0.642-10.761	0.179
**LN status** (Pos *vs* Neg)	3.511	1.074-11.481	0.038
**ER status** (Pos *vs* Neg)	0.561	0.123-2.554	0.455
**PR status** (Pos *vs* Neg)	1.558	0.420-5.775	0.507
**c-erbB-2 status** (Pos *vs* Neg)	1.429	0.422-4.832	0.566
**p53 status** (Pos *vs* Neg)	0.437	0.112-1.703	0.233
**Ki67 status** (Pos *vs* Neg)	0.628	0.120-3.271	0.580
**E-cadherin status** (Pos *vs* Neg)	0.651	0.158-2.678	0.552
**DNMT1 methylation** (M *vs* UM)	1.386	0.337-5.699	0.651

Post, postmenopausal; Pre, premenopausal; LN, lymph node; ER, estrogen receptor; PR, progesterone receptor; Pos, positive; Neg, negative; M, methylated; UM, unmethylated; RR, relative risk; CI, confidence interval.

## Discussion

Promoter methylation, with concurrent changes in histone modifications, is an epigenetic phenomenon which can affect the conformation of chromatin and the accessibility of DNA for transcription factors [13,16]. E2F1 is an important transcription factor and has a highly conserved DNA-binding domain that recognizes a common sequence motif (5′-TTTC[CG]CGC-3′) which is located within the DNMT1 promoter [17]. In this study, we report for the first time that (i) BRCA1-mutated breast cancer displayed a hypermethylated E2F1 motif and promoter region; (ii) the hypermethylated E2F1 motif was negatively correlated with DNMT1 mRNA levels; and (iii) E2F1 is a key transcriptional regulator of DNMT1. The molecular mechanism may involve hypermethylated E2F1 motif-mediated loss of histone modification H3K9ac and transcription factor E2F1 enrichment synergistically inhibiting the transcription of DNMT1. In addition, Additional file 3 showed that no correlation was observed between DNMT1 mRNA or methylated CpG motifs, and H3K9ac or E2F1 enrichment. As shown in Additional file 4, BRCA1 status may also not affect H3K9ac and E2F1 enrichment around the E2F1 motif. These results suggest that abnormal methylation of the E2F1 motif is the initial factor. Interestingly, the synergistic inhibitory effects of hypermethylated E2F1 motif, histone modification H3K9ac and transcription factor E2F1 were primarily observed in cells originating from BRCA1-mutated breast cancer, but transformed cell lines (293 T) and non-mutated breast cancer were insensitive to the loss of H3K9ac and E2F1 enrichment around the E2F1 motif in the core promoter of DNMT1. Accordingly, specific regulatory mechanisms may exist, and DNMT1 expression is likely to be the result of a complex interaction of multiple factors in BRCA1-mutated breast cancer cells. This observation is consistent with previous reports that E2F transcription factors may be key mediators regulating the expression of DNMT1 [17,18], and that DNMT1 was a transcriptional target of BRCA1, as BRCA1 deficiency was associated with decreased levels of DNMT1 [19]. Notably, although the methylation patterns and mRNA expression of DNMT1 showed no significant change in non-BRCA1-mutated breast cancer, the protein expression of DNMT1 is up-regulated. It appears that post-translational modification might be an important method for regulating DNMT1 expression and function. Agoston suggested that the cause of the elevated DNMT1 protein levels could be attributed to an increase in protein half-life in breast cancer [5]. As shown in Additional file 5, global DNA hypomethylation was observed in BRCA1-mutated breast cancer; therefore, it can be speculated that abnormal E2F1 and H3K9ac mediated the decreased expression of DNMT1 might be responsible for the global DNA hypomethylation. It is interesting to note that DNMT1 can be recruited to the promoter of BRCA1, locking in marked suppression of BRCA1 through promoter methylation [20]. Therefore, these data suggest that dynamic cross-talk between DNMT1 and BRCA1 exists in BRCA1-mutated breast cancer.

Promoter hypermethylation is often associated with adverse clinical factors [21]. In line with this, clinicopathological data indicated that the hypermethylated E2F1 motif was significantly associated with histological grade, lymph node, Ki67 and E-cadherin status in BRCA1-mutated breast cancer (Table 1). Moreover, univariate survival analysis demonstrated an association between the hypermethylated E2F1 motif and an increased risk of death. Multivariate survival analysis indicated that lymph node metastasis was an independent prognostic factor for BRCA1-mutated breast cancer patients. This study provides new insights into the causes and prognosis of DNMT1 inactivation in BRCA1-mutated breast cancer.

## Conclusions

Our study identified epigenetic-mediated DNMT1 transcriptional repression, which involved the hypermethylated E2F1 motif-mediated loss of active histone modification H3K9ac and transcription factor E2F1 enrichment in BRCA1-mutated breast cancer. These results will further our understanding as to how the genetic (e.g., BRCA1 mutation) and epigenetic mechanisms (e.g., DNA methylation, histone modifications and transcription factor binding) are jointly involved in the malignant progression of DNMT1-related breast cancer.

## Methods

### Patients and tissue collection

This study was approved by the Institutional Review Board at China Medical University. Eighty-five invasive ductal carcinomas from BRCA1 mutation carriers and fifteen invasive ductal carcinomas from non-BRCA1 mutation carriers were enrolled between 2007 and 2009; all patients gave informed consent. Fresh breast cancer and adjacent normal breast tissues were obtained at the time of primary surgery before any chemotherapy or radiotherapy had been administered. Hematoxylin and eosin staining of the samples for histopathological diagnosis and grading were performed by three staff pathologists using the Nottingham Combined Histologic Grade. The tumor stages were classified according to the National Comprehensive Cancer Network guidelines. All patients were screened for BRCA1 mutations by multiplex polymerase chain reaction with complete sequence analysis as previously reported [22-24]. Their characteristics are given in Table 1.

### Cell culture, lentiviral infection and cell proliferation assay

Detailed isolation and cultivation protocols were established as previously described [25]; breast cancer and normal cells were maintained in CnT-27 mammary epithelium medium (CELLnTEC, Bern, Switzerland). Human 293 T cells were maintained in Dulbecco’s modified Eagle’s medium with 10% fetal bovine serum (Invitrogen, CA USA). Lentiviral vectors expressing short hairpin RNAs (shRNA) against BRCA1 (NM_007299) were obtained from Genechem Co., Ltd (Shanghai, China), and synthesized as follows: Forward: 5-CCGGAACCTGTCTCCACAAAGTGTGCTCGAGCACACTTTGTGGAGACAGGTTTTTTTG, and Reverse: 5-AATTCAAAAAAACCTGTCTCCACAAAGTGTGCTCGAGCACACTTTGTGGAGACAGGTT. The non-silencing siRNA sequence (TTCTCCGAACGTGTCACGT) was used as a negative control. For overexpression of BRCA1, the open reading frame of BRCA1 (NM_007299) was cloned into the lentiviral vector GV287 (Ubi-MCS-3FLAG-SV40-EGFP) (GeneChem). The shRNA lentiviral particles of GCN5 (sc-37946-V), PCAF (sc-36198-V) and E2F1 (sc-29297-V) were purchased from Santa Cruz Biotechnology (CA, USA). Transfections were performed using the polybrene and enhanced infection solution (Genechem) according to the manufacturer’s recommended protocol, the knowdown effiency for the double-knockdown and triple-knockdown were shown in Additional file 6. After 48 hours of infection, cell proliferation was determined using the Cell-Light™ EdU Apollo®643 *In Vitro* Imaging Kit (Ribobio, Guangzhou, China) following the instructions provided by the manufacturer.

### DNA methylation analysis

Genomic DNA from the breast cancer and adjacent normal breast tissues was extracted using a TIANamp Genomic DNA kit (Tiangen Biotech, Beijing, China). Sodium bisulfite conversion, polymerase chain reaction (PCR) amplification and general experimental procedure were performed as previously described [22]. The specific primer sequences for bisulfite sequencing and methylation-specific PCR are described in Additional file 7. Global DNA methylation was measured as previously described [26].

### Real-time PCR and immunohistochemistry analysis

Real-time PCR and immunohistochemistry were performed as previously described [22]. The specific primer sequences for real-time PCR are listed in Additional file 7. The primary antibody for immunohistochemistry was rabbit anti-DNMT1 of human origin, and are listed in Additional file 8 (1:200; Santa Cruz Biotechnology).

### Chromatin immunoprecipitation (CHIP), site-directed mutagenesis, transfection and dual-luciferase reporter assay

CHIP, site-directed mutagenesis, transfection and dual-luciferase reporter assays were performed as previously described [27]. The specific primer sequences for site-directed mutagenesis and CHIP are provided in Additional file 7. The specific antibodies for CHIP are provided in Additional file 8.

### Co-immunoprecipitation (CO-IP) and immunoblotting

CO-IP was performed using an immunoprecipitation kit (Invitrogen) according to the manufacturer’s recommended protocol. Then, the cell lysates and immunoprecipitates were analyzed by immunoblotting. The specific antibodies for CO-IP and immunoblotting are provided in Additional file 8.

### Circular dichroism (CD) spectra

The CD spectra were obtained on a Jasco J-810 spectropolarimeter at 25°C using a 0.1 cm path length cell; data were collected with a 2 nm slit width from 350 to 200 nm at 0.5 nm intervals and averaged over three scans. CD experiments were carried out on DNA samples (5 μM) using a buffer containing 0.2 M phosphate buffer (pH 7.0) in the presence of 100 mM Na^+^ or K^+^. The DNA samples were annealed by heating to 95°C for 5 min followed by cooling to room temperature over 10 h before analysis. The DNA sequence is as follows: 5-CCCCTCCCCATCGGTTTC“C (CH_3_ or non-CH_3_)” GCGCGAAAAGCCGGGGCGCC, and was synthesized by Sangon Biotech Ltd (Shanghai, China).

### Statistical analysis

Regression analysis was used to examine the possible relationship between DNMT1 mRNA or protein levels, and the status of promoter methylation. The association between clinicopathological features and DNMT1 promoter methylation was determined using the Fisher’s exact test. Univariate analysis of survival was performed using the Kaplan-Meier method. Multivariate Cox regression analysis was performed to identify the independent prognostic factors for overall survival. The data are presented as means ± SD. Statistical differences in the data were evaluated by Student’s *t* test or one-way ANOVA as appropriate, and were considered significant at *P* < 0.05.

## Abbreviations

CD: Circular dichroism; CHIP: Chromatin immunoprecipitation; CI: Confidence interval; CO-IP: Co-immunoprecipitation; DNMT1: DNA methyltransferase 1; E2F1: E2F transcription factor 1; ER: Estrogen receptor; LN: Lymph node; M: Methylated; Neg: Negative; PCR: Polymerase chain reaction; Pos: Positive; Post: Postmenopausal; PR: Progesterone receptor; Pre: Premenopausal; RR: Relative risk; shRNA: Short hairpin RNAs; UM: Unmethylated.

## Competing interests

The authors declare that they have no competing interests.

## Authors’ contributions

DL and QY conceived of the study, participated in its design and drafted the manuscript. DL, FFB and JMC carried out data acquisition and interpretation. CC and BL participated in the design of the study and performed the statistical analysis. All authors read and approved the final manuscript.

## Supplementary Material

Additional file 1 Expression levels of DNMT1 in non-mutated and BRCA1-mutated breast cancer and their adjacent normal breast tissues. Click here for file

Additional file 2 Comparative analysis of histone modification and E2F1 enrichment around the E2F1 motif between non-BRCA1-mutated breast cancer and their adjacent normal breast tissues. Click here for file

Additional file 3 Correlation between the H3K9ac or E2F1 enrichment, and +182 site methylation or DNMT1 expression in BRCA1-mutated breast cancer and their adjacent normal breast tissues. Click here for file

Additional file 4 H3K9ac or E2F1 enrichment after silencing or overexpression of BRCA1 in 293 T cells, and primary non-mutated and BRCA1-mutated breast cancer and their normal breast cells. Click here for file

Additional file 5 Compared of global DNA methylation levels between BRCA1-mutated breast cancer and their adjacent normal breast tissues. Click here for file

Additional file 6 Knowdown effiency for the double-knockdown and triple-knockdown in BRCA1-mutated breast cancer cells. Click here for file

Additional file 7 Primers used in this study. Click here for file

Additional file 8 List of commercial antibodies. Click here for file
