# Supplementary material for: Regulation of DNA methyltransferase 1 transcription in BRCA1-mutated breast cancer: a novel crosstalk between E2F1 motif hypermethylation and loss of histone H3 lysine 9 acetylation
Source: Mol Cancer. 2014 Feb 6;13:26. doi: 10.1186/1476-4598-13-26 (PMC3936805; doi:10.1186/1476-4598-13-26)
Supplement: Additional file 1 — Expression levels of DNMT1 in non-mutated and BRCA1-mutated breast cancer and their adjacent normal breast tissues. [file 1476-4598-13-26-S1.pdf]

Additional file 1

Expression levels of DNMT1 in non-mutated and BRCA1-mutated breast cancer and their adjacent normal breast tissues

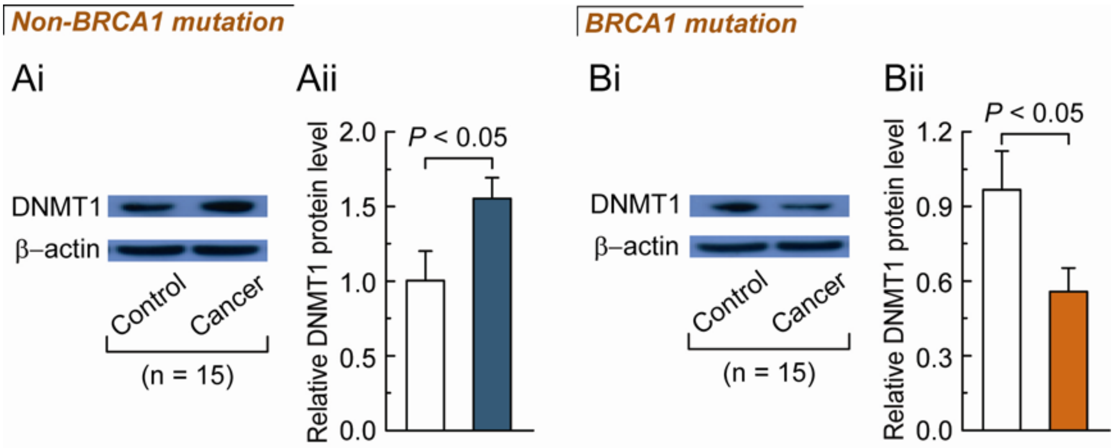

Ai and Bi, western blot analysis of DNMT1 in non-mutated and BRCA1-mutated breast cancer, compared to their adjacent normal tissue, respectively. The blot is representative of three separate experiments (each group, n = 15); Aii and Bii, summary of the results from Ai and Bi, respectively. Bar graphs show mean ± SD.
