# Supplementary material for: Regulation of DNA methyltransferase 1 transcription in BRCA1-mutated breast cancer: a novel crosstalk between E2F1 motif hypermethylation and loss of histone H3 lysine 9 acetylation
Source: Mol Cancer. 2014 Feb 6;13:26. doi: 10.1186/1476-4598-13-26 (PMC3936805; doi:10.1186/1476-4598-13-26)
Supplement: Additional file 2 — Comparative analysis of histone modification and E2F1 enrichment around the E2F1 motif between non-BRCA1-mutated breast cancer and their adjacent normal breast tissues. [file 1476-4598-13-26-S2.pdf]

Additional file 2

Comparative analysis of histone modification and E2F1 enrichment around the E2F1 motif between non-BRCA1-mutated breast cancer and their adjacent normal breast tissues

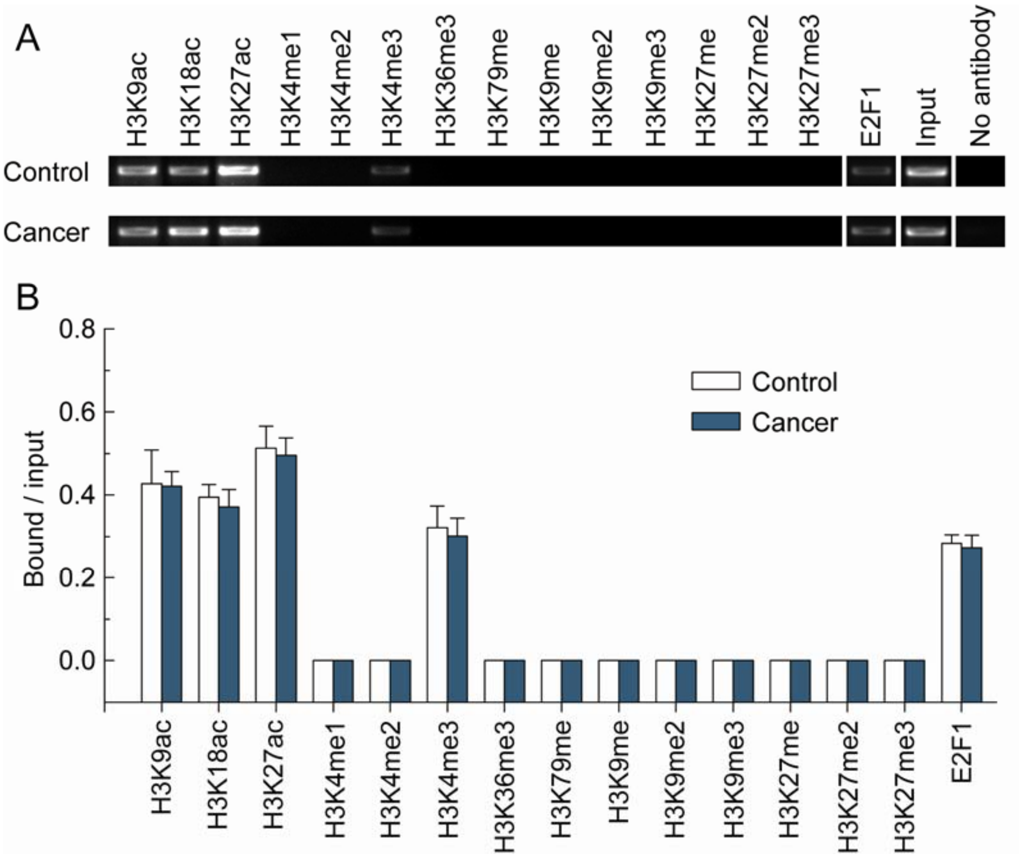

A, chromatin immunoprecipitation was performed using antibodies to H3K9Ac, H3K18Ac, H3K27Ac, H3K4me1, H3K4me2, H3K4me3, H3K36me3, H3K79me, H3K9me, H3K9me2, H3K9me3, H3K27me, H3K27me2, H3K27me3 and E2F1. PCR was performed for regions within the CpG islands and around the E2F1 motif. A negative control without antibodies is included for comparison. The blot is representative of three separate experiments. B, representative results of 15 primary non-BRCA1-mutated breast cancer and their normal breast cells are shown. Bar graphs show mean ± SD.
