# Supplementary material for: Regulation of DNA methyltransferase 1 transcription in BRCA1-mutated breast cancer: a novel crosstalk between E2F1 motif hypermethylation and loss of histone H3 lysine 9 acetylation
Source: Mol Cancer. 2014 Feb 6;13:26. doi: 10.1186/1476-4598-13-26 (PMC3936805; doi:10.1186/1476-4598-13-26)

Additional file 3

Correlation between the H3K9ac or E2F1 enrichment, and +182 site methylation or DNMT1 expression in BRCA1-mutated breast cancer and their adjacent normal breast tissues

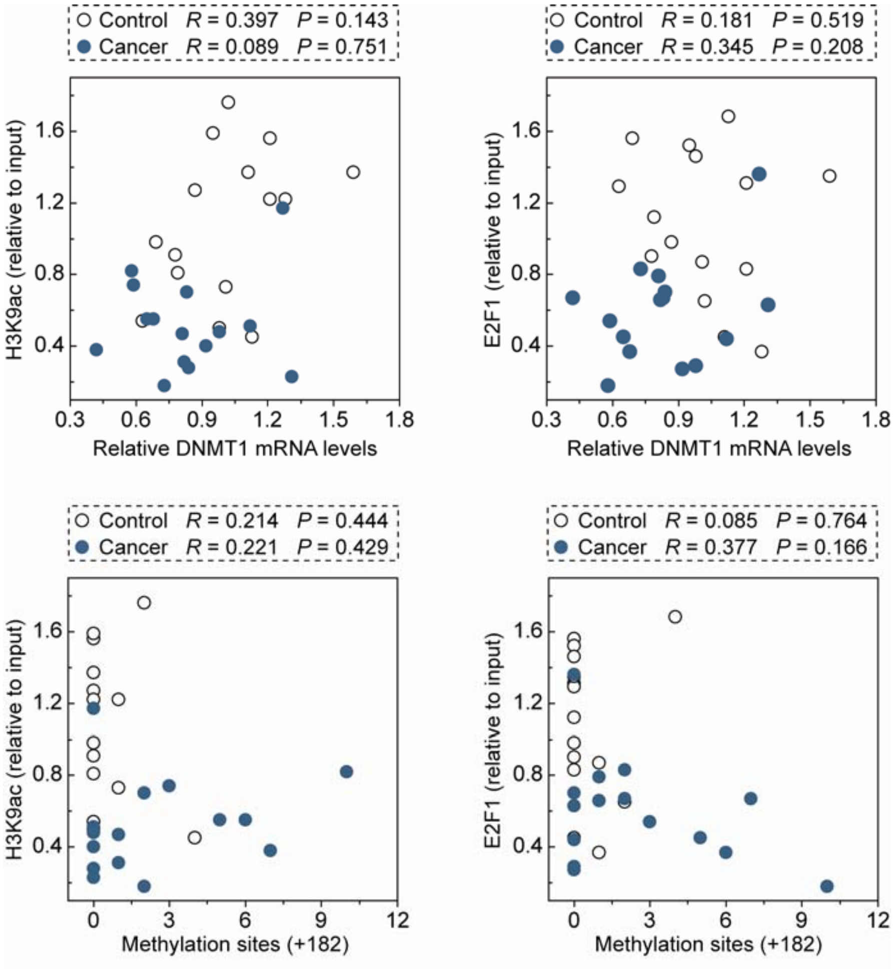

Supplement: Additional file 3 — Correlation between the H3K9ac or E2F1 enrichment, and +182 site methylation or DNMT1 expression in BRCA1-mutated breast cancer and their adjacent normal breast tissues. [file 1476-4598-13-26-S3.pdf]
