# Supplementary material for: Regulation of DNA methyltransferase 1 transcription in BRCA1-mutated breast cancer: a novel crosstalk between E2F1 motif hypermethylation and loss of histone H3 lysine 9 acetylation
Source: Mol Cancer. 2014 Feb 6;13:26. doi: 10.1186/1476-4598-13-26 (PMC3936805; doi:10.1186/1476-4598-13-26)
Supplement: Additional file 4 — H3K9ac or E2F1 enrichment after silencing or overexpression of BRCA1 in 293 T cells, and primary non-mutated and BRCA1-mutated breast cancer and their normal breast cells. [file 1476-4598-13-26-S4.pdf]

Additional file 4

H3K9ac or E2F1 enrichment after silencing or overexpression of BRCA1 in

293T cells, and primary non-mutated and BRCA1-mutated breast cancer and their normal breast cells

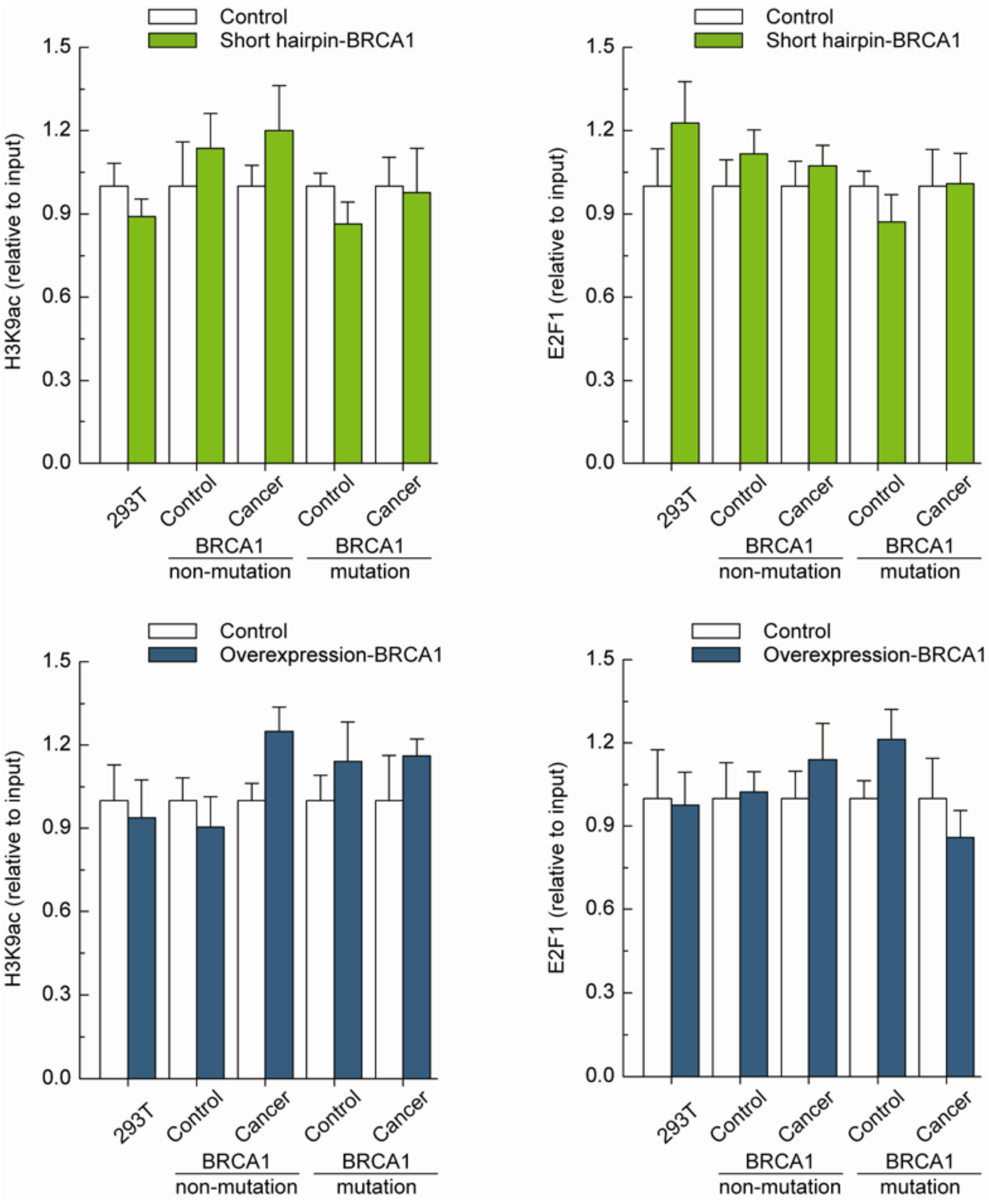

Each experiment was repeated four times for 293T cells and primary breast cells of each patient (n = 15). Bar graphs show mean ± SD.
