# Supplementary material for: Regulation of DNA methyltransferase 1 transcription in BRCA1-mutated breast cancer: a novel crosstalk between E2F1 motif hypermethylation and loss of histone H3 lysine 9 acetylation
Source: Mol Cancer. 2014 Feb 6;13:26. doi: 10.1186/1476-4598-13-26 (PMC3936805; doi:10.1186/1476-4598-13-26)
Supplement: Additional file 5 — Compared of global DNA methylation levels between BRCA1-mutated breast cancer and their adjacent normal breast tissues. [file 1476-4598-13-26-S5.pdf]

Additional file 5

Compared of global DNA methylation levels between BRCA1-mutated breast cancer and their adjacent normal breast tissues

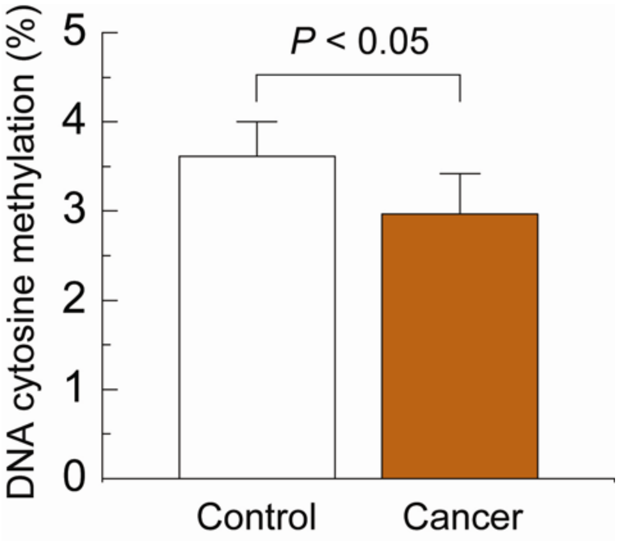

Each experiment was repeated three times for each tissues (n = 15). Bar graphs show mean ± SD.
