# Supplementary material for: Regulation of DNA methyltransferase 1 transcription in BRCA1-mutated breast cancer: a novel crosstalk between E2F1 motif hypermethylation and loss of histone H3 lysine 9 acetylation
Source: Mol Cancer. 2014 Feb 6;13:26. doi: 10.1186/1476-4598-13-26 (PMC3936805; doi:10.1186/1476-4598-13-26)
Supplement: Additional file 6 — Knowdown effiency for the double-knockdown and triple-knockdown in BRCA1-mutated breast cancer cells. [file 1476-4598-13-26-S6.pdf]

Additional file 6

Knowdown effiency for the double-knockdown and triple-knockdown in BRCA1-mutated breast cancer cells

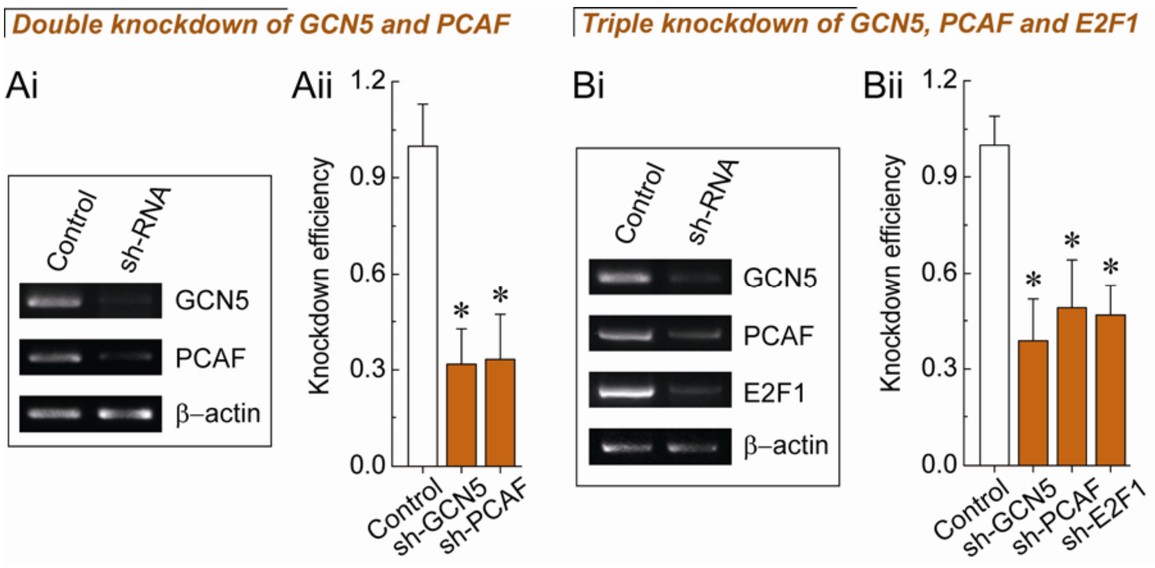

Ai and Bi, RT-PCR showing GCN5, PCAF and E2F1 levels before and after knockdown by shRNAs, and normalized to β-actin expression. Aii and Bii, the results from three independent experiments are represented as mean ± SD.
