# Supplementary material for: Regulation of DNA methyltransferase 1 transcription in BRCA1-mutated breast cancer: a novel crosstalk between E2F1 motif hypermethylation and loss of histone H3 lysine 9 acetylation
Source: Mol Cancer. 2014 Feb 6;13:26. doi: 10.1186/1476-4598-13-26 (PMC3936805; doi:10.1186/1476-4598-13-26)
Supplement: Additional file 7 — Primers used in this study. [file 1476-4598-13-26-S7.pdf]

Additional file 7

Primers used in this study

Gene	Primers	Description
DNMT1-BSP-F	5’- TTTAGAGTAGGTGTAATTAT	Methylation analysis for
DNMT1-BSP-R	5’- CCAAACTAAAATCAAAAACC	DNMT1 (Round I)
DNMT1-BSP-F	5’- TGGAATTGAGGATTTTATTTAAGG	Methylation analysis for
DNMT1-BSP-R	5’- TAATTTCTAACCACCAAAAAACTAC	DNMT1 (Round II)
DNMT1-MSP-MF	5’- AGTAAATTGTGGAGTTTGGATGAGTTTA	Methylation analysis for
DNMT1-MSP-MR	5’- AACACAAACACCCCAACTTTTCACACG	DNMT1
DNMT1-MSP-UF	5’- AGTAAATTGTGGAGTTTGGATGAGTTTA	Methylation analysis for
DNMT1-MSP-UR	5’- AACACAAACACCCCAACTTTTCACACA	DNMT1
DNMT1-RTP-F	5’- TACCTGGACGACCCTGACCTC	Real-time PCR for DNMT1
DNMT1-RTP-R	5’- CGTTGGCATCAAAGATGGACA	
GAPDH-RTP-F	5’- AGGTGAAGGTCGGAGTCA	Real-time PCR for GAPDH
GAPDH-RTP-R	5’- GGTCATTGATGGCAACAA	
GCN5-SQP	Santa Cruz Biotech	Semi-quantitative PCR for
	GCN5 (h)-PR: sc-37946-PR	GCN5
PCAF-SQP	Santa Cruz Biotech	Semi-quantitative PCR for
	PCAF (h)-PR: sc-36198-PR	PCAF
E2F1-SQP	Santa Cruz Biotech	Semi-quantitative PCR for
	E2F-1 (h)-PR: sc-29297-PR	E2F1
DNMT1-ChIP-RTP-F	5’- TTCTGCACAGGGTATCGCCT	ChIP for DNMT1 (Detection
DNMT1-ChIP-RTP-R	5’- ATCTCGGAGGCTTCAGCAGA	by real-time PCR)
DNMT1-ChIP-SQP-F	5’- GTATCGCCTCTCTCCGTTTG	ChIP for DNMT1 (Detection
DNMT1-ChIP-SQP-R	5’- GGACGGCCAGTGTGGGCACC	by semi-quantitative PCR)
DNMT1-Mutation-F	5’- TCCCCATCGGTTTCTGCGCGAAAAGCCGG	A point mutation at the site
DNMT1-Mutation-R	5’- CCGGCTTTTCGCGCAGAAACCGATGGGGA	of +182 (C to T)

List of abbreviations used: BSP, Bisulfite-sequencing PCR; MSP, Methylation-specific PCR; RTP, Real-time PCR; SQP, Semi-quantitative PCR; ChIP, Chromatin immunoprecipitation; F, Forward primer; R, Reverse primer; M, methylated; U, unmethylated.
