# Supplementary material for: Regulation of DNA methyltransferase 1 transcription in BRCA1-mutated breast cancer: a novel crosstalk between E2F1 motif hypermethylation and loss of histone H3 lysine 9 acetylation
Source: Mol Cancer. 2014 Feb 6;13:26. doi: 10.1186/1476-4598-13-26 (PMC3936805; doi:10.1186/1476-4598-13-26)
Supplement: Additional file 8 — List of commercial antibodies. [file 1476-4598-13-26-S8.pdf]

Additional file 8

List of commercial antibodies

Antibody	Company (catalog number)	Description
H3K9Ac	Abcam (ab4441)	ChIP
H3K18Ac	Abcam (ab1191)	ChIP
H3K27Ac	Millipore (07-360)	ChIP
H3K4me1	Abcam (ab8895)	ChIP
H3K4me2	Abcam (ab7766)	ChIP
H3K4me3	Abcam (ab8580)	ChIP
H3K36me3	Abcam (ab9050)	ChIP
H3K79me	Abcam (ab2886)	ChIP
H3K9me	Abcam (ab9045)	ChIP
H3K9me2	Millipore (07-441)	ChIP
H3K9me3	Abcam (ab8898)	ChIP
H3K27me	Millipore (07-448)	ChIP
H3K27me2	Millipore (07-452)	ChIP
H3K27me3	Millipore (07-449)	ChIP
E2F1	Millipore (17-10061)	ChIP, IP, WB
GCN5	Santa Cruz (sc-20698)	IP, WB
PCAF	Santa Cruz (sc-13124)	IP, WB
DNMT1	Santa Cruz (sc-20701)	IHC

List of abbreviations used: ChIP, Chromatin immunoprecipitation; IP, Immunoprecipitation; WB, Western blotting; IHC, Immunohistochemistry.
